# YIM-O20. Standardization and performance of cone beam ct scan for the evaluation of linear scleroderma of the face

**DOI:** 10.1186/1546-0096-12-S1-Y2

**Published:** 2014-09-17

**Authors:** Chiara Di Giovanni, Roberta Culpo, Fabio Vittadello, Sabina Trainito, Stefano Puggina, Giorgia Martini, Francesco Zulian

**Affiliations:** 1 Department of Pediatrics, University of Padua, Padua, Italy; 2 Orthopedic Rehabilitation, Department of Neurosciences, University of Padua, Padua, Italy; 3 Euromedic Group – Iniziativa Medica, Diagnostic imaging, Padova, Italy

## Introduction

Up to now, standardized and validated methods for monitoring linear scleroderma of the face are still lacking. Therefore, it is difficult to assess, in these patients, the disease progression both for therapeutic purposes and maxillofacial surgery procedures. The Cone Beam Computed Tomography (CBCT) is an imaging technique that has good sensitivity for both soft and the bony tissues, is fast to be performed, does not require sedation and the irradiation is 50 times lower than traditional CT scan.

## Objectives

We evaluated the performance of Cone Beam CT scan for evaluation of linear scleroderma of the face.

## Methods

The study was conducted in two phases. Five CBCT transverse sections were identified, in order to describe the entire face volume: mental symphysis (MS), mandibular foramen (MF), maxillary sinus (MS), mandibular condyle (MC) and supraorbital ridge (SR). From the intersection axes an origin point was generated and from this one, 30° and 60° lines, crossing bony and soft structures, were drawn. For each given degree, the total thickenss, the soft tissue thickness, and the inner skeletal thickness, were measured. Sixty measures were therefore evaluated for each patient by using the software Onis 2.4 free edition. The intra- and inter-operator correlation were tested, ICC values were interpreted as follows: > 0.7 excellent, 0.40–0.75 fair to good and <0.40 as poor.

## Results

The lack of reproducible bony landmarks allowed us to discard two transverse sections: the supraorbital ridge and the mandibular symphysis, which represent the more external sections of the face. Three judges evaluated 5 patients’ CBCTs twice, with a time interval between the first and second analysis of one month. The same evaluators analyzed 10 patients’ CBCTs singularly and blindly to the others and to the disease site. The intra-operator concordance resulted optimal in almost all cases, varying from a minimum of 0.70 to a maximum of 1. The inter-operator concordance between judges was statistically significant in the 75% of cases, with an ICC ranging between 0.40 and 0.92. About 25% of the parameters had an optimal ICC (> 0.75). The best performances were obtained at the level of the MF and MC planes.

## Conclusion

CBCT has shown to be a reliable method to assess skin and bone lesions in patients with linear scleroderma of the face. It is fast to be done, safe and reproducible. While it is not applicable for frontal lesions, it provides a reliable assessment in the remaining parts of the face. A prospective validation to confirm its relevance in evaluating the disease progression is on going.

## Disclosure of interest

None declared

